# Large Language Models in Surgery: Promise, Pitfalls, and Practical Use

**DOI:** 10.3389/jaws.2026.16349

**Published:** 2026-03-26

**Authors:** Danette T. Denham, Colin Y. Wang, Emil Maric, Lucy R. Hinton, B. Todd Heniford

**Affiliations:** Division of Gastrointestinal and General Surgery, Department of Surgery, Endeavor Health, Evanston, IL, United States

**Keywords:** academic research, artificial intelligence (AI), large language models, patient outcomes, surgery

## Abstract

**Background:**

Large Language Models (LLMs) represent a transformative advancement in artificial intelligence (AI) with rapidly expanding applications in medicine. While AI-related medical publications increased 36-fold between 2000–2022, practical guidance for surgeons remains limited. This mini-review delineates pragmatic applications of LLMs in surgical practice while addressing key limitations, implementation considerations, and ethical considerations.

**Methods:**

We reviewed contemporary LLM platforms and their integration into clinical workflows, patient communication, surgical research and academic writing, evaluating both benefits, constraints and risk mitigation relevant to practicing surgeons.

**Findings:**

LLMs demonstrate significant utility across multiple domains. In clinical workflows, ambient documentation and chart summarization may reduce documentation burden and support rapid synthesis of complex patient data. For patient communication, these tools can simplify complex medical information, tailor or translate patient instructions to appropriate reading levels or languages, and generate empathetic responses to patient messages with improved efficiency. In research, LLMs assist with literature summarization, study design optimization, and risk of bias assessment in RCT, allowing surgeons to focus on higher-level scientific reasoning. Despite promising applications, several constraints demand attention. Effective prompting requires specific techniques including clear clinical objectives, explicit instructions, and iterative refinement. LLM outputs require verification to prevent “hallucinations” - fabricated or inaccurate information. Protected health information (PHI) must never be entered into public LLM platforms to maintain HIPAA compliance. Liability frameworks for AI-generated errors remain ambiguous, with unclear responsibility deferred amongst providers, institutions, and developers.

**Conclusion:**

LLMs offer surgeons valuable tools for enhancing workflow efficiency and patient communication when deployed with appropriate oversight. Success requires understanding prompt engineering principles, maintaining rigorous fact-checking protocols, protecting patient privacy, and recognizing that human judgment remains irreplaceable in clinical decision-making.

## Introduction

Artificial Intelligence (AI) represents one of the most disruptive technological advances of the modern era, with the potential to impact nearly every aspect of human life. Within the field of medicine, interest in AI has increased exponentially, with a 36-fold increase in AI-related medical publications between 2000–2022, growing from approximately 8,500 publications to over 307,000 [1]. Large language models (LLMs) are a subclassification of AI that use transformer-based neural networks trained on extensive datasets to learn semantic and statistical patterns of tokens, allowing interpretation and generation of human-like responses to prompts without task-specific training [2, 3].

The first widely recognized LLM was Generative Pre-trained Transformer (GPT)-3.5, released in November 2022 by OpenAI, followed by GPT-4 in March 2023 [4]. Subsequently, comparable models such as Bard [5] (now Google Gemini) and Bing Chat [6] (now Microsoft CoPilot) have entered the market (Table 1). Proposed clinical applications for these revolutionary LLMs span a diverse range of functions, including improving diagnostic capabilities, predicting outcomes, reducing clinical documentation burden, improving medical education, and filtering the expanding research literature. However, persistent uncertainties regarding the accuracy, reproducibility, and ethical governance of these systems impede their ubiquitous clinical adoption [7].

**TABLE 1:** Overview of relevant AI with parent company, price, and HIPAA compliance.

LLM name	Parent company	Cost	HIPAA compliance
Abridge [9]	None [9]	Must contact company for estimate [9]	Yes [70].
ChatGPT^a^ [71]	OpenAI [71]	Pro: $20/month Plus: $200/month [72]	OpenAI for healthcare products are HIPAA compliant, not ChatGPT single subscriptions [73].
Claude^a^ [74]	Anthropic [74]	Pro: $20/month Max:$100/month [75]	Healthcare option HIPAA-ready, not single subscriptions [76].
Copilot^a^ [77] (formally bing chat)	Microsoft [77]	Personal: $9.99/month Premium: $19.99/month [78]	HIPAA compliance with copilot studio, a feature available to organizations [79].
Gemini^a^ [80] (formally bard)	Alphabet inc [80]	Pro: $19.99/month Ultra [2]: $124.99/month [81]	Compliance with HIPAA accessible through google workspace or BAA [82].
Grok^a^ [83]	xAI [83]	SuperGrok: $30/month SuperGrok heavy: $300/month [84]	Can support HIPAA under BAA [85].
OpenEvidence [86]	None [86]	Free for US healthcare professionals with NPI [86]	Yes [87].

BAA: Business Association Agreement; NPI: National provider identifier.

^a^ Free version available for public

Despite expanding interest in medical LLMs, practical guidance tailored to surgical workflows remains limited. At the same time, physicians generally express positive attitudes toward adopting AI tools when they are feasible and clinically useful [8]. The aim of this review is to delineate clear and pragmatic uses for surgeons with currently available LLM platforms. The limitations of AI use within medicine will be evaluated, including the ethical considerations with LLM deployment in surgical practice.

### LLM Integration With Electronic Health Record Systems

For many physicians, their initial clinical experience with LLMs will occur through their electronic health record (EHR) system. These tools fall into two categories: those developed natively by EHR vendors such as Epic or Oracle Health and third-party applications designed to integrate seamlessly with existing EHR platforms. The integration model offers distinct advantages, including smoother incorporation into established clinical workflows and institutional vetting of data security practices and regulatory compliance. However, this approach also introduces access barriers, as many tools require institutional licenses rather than individual subscriptions. Despite these constraints, LLM-integrated EHR tools are rapidly proliferating across healthcare settings and represent an important frontier in clinical AI adoption.

Peer-reviewed evidence for these commercial tools remains limited due to their proprietary nature and rapid development cycles. Much of the available information regarding features, performance metrics, and clinical benefits originates from vendor marketing materials, which should be evaluated with appropriate skepticism. The following discussion provides a sampling of currently available tools to illustrate the landscape of LLM integration in EHR systems and is not intended as an endorsement of any particular vendor or product. Healthcare organizations and individual clinicians should conduct thorough due diligence, including review of any available independent validation studies and consideration of institutional needs, before adopting these technologies.

### Ambient Clinical Documentation

Perhaps the most widely adopted LLM application in EHRs is ambient clinical documentation, which uses AI to automatically generate clinical notes from recorded patient-clinician conversations. Third-party platforms such as Abridge [9], Nuance DAX Copilot [10], and DeepScribe [11] integrate with major EHR systems including Epic, Cerner/Oracle Health, and Athenahealth. A multicenter study by Olson et al. demonstrated that ambient AI scribes reduced clinician burnout from 51.9% to 38.8% within 30 days, while decreasing both note-related cognitive task load by 2.64 points on a 10-point scale and after-hours documentation time by 0.90 h [12]. A separate study by Moura et al. found that hybrid ambient clinical documentation combining generative AI with virtual scribes reduced “work outside of work” by 41.7% and improved financial productivity by 12.1% within 50 days [13]. DAX Copilot’s has demonstrated ability to provide accurate, thorough inpatient note taking with succinct synthesis of information whilst limiting hallucinations and bias [14].

EHR vendors have also developed native ambient documentation capabilities. Epic’s ambient solution [15] and Oracle Health’s next-generation EHR [16] have incorporated ambient documentation features into their platforms. Additional entrants (Athelas AIR [17] and eClinicalWorks’ Sunoh [18]) offer complete EHR platforms with built-in ambient AI functionality. Beyond real-time note generation, these tools often include autonomous medical coding capabilities that automatically suggest CPT and ICD-10 codes from the generated clinical notes, further streamlining administrative workflows and reducing administrative burden. A vendor-reported study facilitated by Microsoft showed a 3.4% increased level of service (LOS) when DAX Copilot for EPIC was used [10], but independent validation is limited.

### Clinical Intelligence and Patient Data Aggregation

LLMs are increasingly being deployed within EHRs to synthesize complex patient data and generate actionable clinical insights. Note summarization features create context-specific summaries of patient charts tailored to different care settings, such as emergency department triage versus annual physicals [15]. These summaries include citations linking back to source information, allowing clinicians to quickly grasp a patient’s status without manually reviewing multiple chart entries.

Athenahealth’s Clinically Inferred Diagnosis feature uses multi-dimensional data analysis to suggest potential diagnoses and identify care gaps based on historical patient health data, medication lists, and past encounters [19]. Navina’s clinical intelligence platform reconciles historical patient records with real-time data streams from multiple sources including labs, imaging, and clinical notes to support value-based care initiatives [20]. The platform identifies open care gaps and coding opportunities during patient encounters, integrating with ambient AI tools to align live patient dialogue with historical records.

PatientKeeper, now part of Commure, provides AI-driven 12-h patient summaries that highlight significant changes, treatments, and key patient data, along with a conversational AI chat feature that allows clinicians to quickly retrieve specific information without time-consuming manual searches [21]. Oracle Health’s next-generation EHR incorporates Oracle Health Data Intelligence, which continuously integrates patient data from clinical, claims, social determinants, and pharmacy sources to deliver real-time insights for personalized care planning [16]. These tools aim to reduce the cognitive burden of information foraging while supporting more informed clinical decision-making at the point of care.

### Revenue Cycle Management Automation

LLM’s are transforming revenue cycle management by automating traditionally labor-intensive billing and coding tasks. Commure’s Autonomous Coding platform integrates with Epic, Cerner/Oracle Health, MEDITECH, and over 30 other EHR systems, automatically generating CPT codes, ICD-10 diagnoses, and modifiers directly from clinical documentation [21]. Epic’s “Penny” AI agent assists with billing code suggestions and generates appeal letters for denied insurance claims [15]. These tools address the growing complexity of medical coding.

By leveraging ambient documentation outputs, autonomous coding systems can achieve high accuracy rates while reducing manual review burden. The integration of these tools with upstream documentation platforms creates an end-to-end workflow from patient encounter to claim submission, potentially improving first-pass claim acceptance rates and reducing days in accounts receivable. However, implementation success depends on robust data flow from the EHR, documentation quality, and alignment between AI vendors and institutional coding practices.

### Assisted Practice in the Clinical Setting

Current LLMs may assist clinicians in perioperative risk stratification; accurately predicting ICU admission, unplanned hospital admission, and mortality using real-world EHR data. The models performed well with categorical outcomes but were less accurate with continuous numerical predictions such as length of stay [22]. ChatGPT showed superior performance over competing LLMs in use of clinical risk assessments tools such as Charlson Comorbidity Index to estimate patient clinical risk [23]. ChatGPT can be used to provide recommendations for thromboembolic prophylaxis with reasonable accuracy, which can help to mitigate surgical risk whilst reducing cognitive load of the treating team [24].

## Patient Communication

Physician-patient communication is foundational component of medical care and is associated with patient satisfaction, outcomes, and medicolegal risk [1, 3, 25]. LLM’s have the potential to streamline patient communication by drafting responses to patient questions, editing existing patient material, and translating medical information into understandable content [26]. As patient access to clinical notes, laboratory results, and imaging reports becomes more common, discordance between clinical language, and patient understanding can increase anxiety and confusion. Multiple studies have shown that LLMs can produce generally accurate summaries of radiology reports, whilst maintaining important diagnostic information. Similarly, LLMs can be used to convert discharge summaries to more patient-friendly formats [27, 28].

Health literacy is an important social determinant of health, with poor literacy associated with worse health outcomes [29, 30]. Written educational materials facilitate patient understanding and enable patients to revisit information beyond the clinical encounter; however they are often well above readability levels appropriate for the general population (8th grade or less) [31, 32]. ChatGPT-4 has been to shown to reliably “Rewrite the following at a 6th-grade reading level” resulting in patient material with improved readability [32]. Similarly, Abreu and colleagues evaluated patient-facing cancer information from 34 NCCN-affiliated institutions and found that ChatGPT-4 reliably improved readability from approximately a college-freshman level to roughly a ninth-grade level while preserving accuracy and overall quality [33]. These findings support the use of LLMs as an editing layer for expert-generated content to reduce health-literacy barriers without materially degrading informational fidelity.

Additionally, Spanish-speaking patients represents a substantial portion of the US population, and many prefer postoperative instructions in their native language [34, 35]. LLMs have been shown to more accurately translate discharge instructions in Spanish and Portuguese when compared to Google Translate [36]. When using utilizing LLM generative writing capabilities for translation, outputs should be reviewed for accuracy and to ensure they adhere to institutional policies.

Patient messaging can be a major time burden. One study noted physicians spend an average of 2.3 min responding to each patient message [37]. LLMs can draft responses that clinicians then review and edit, potentially saving time while maintaining quality. In comparative evaluations, LLM-generated responses having similar accuracy, empathy but significantly higher word count per question answered [38, 39]. Answers to patient questions can be increased with accurate LLM prompting, such as instructing the LLM to simulate an experienced orthopedic surgeon [40]. Used thoughtfully and with clinician oversight, this technology creates time-efficient, clear patient communication whilst concurrently improving patient understanding.

## Research

The integration of LLMs into academic research workflows has grown rapidly. Recent analyses suggest a marked increase in AI assistance in manuscripts and preprints [41], and surveys of clinical researchers report leveraging LLMs for tasks perceived to improve efficiency (question formulation, literature review, data summarization, manuscript editing), but persistent concerns regarding accuracy, bias, and transparency remain [42].

### Literature Review and Evidence Synthesis

Generative AI has been proposed as a support mechanism in evidence synthesis workflows, including strategy generation and screening assistance. Studies evaluating LLM performance for real-time systematic search tasks reveal mixed performance. While models like ChatGPT can generate structured search queries or assisted screening prompts, current evidence indicated these models frequently miss large proportions of relevant studies or may provide irrelevant outputs when used as standalone search agents [43]. Nonetheless, ChatGPT-4 and Gemini were able to draft literature search syntax for a systematic literature review [44]. The ability to generate search syntax streamlines the review process while preserving investigator authority over article selection.

### AI in Study Design

Emerging research suggests that when guided by expert oversight, AI-generated frameworks can match conventional RCT design criteria, with the potential to improve representativeness and generalizability of trial populations [45]. ChatGPT and Claude demonstrate high rates of accuracy in structured tasks, such as assessing the Risk of Bias in RCTs, allowing for more efficient appraisal when paired with human verification of study validity [46]. ChatGPT can be assistive in generating queries for systematic review with high precision [47]. When prompted, ChatGPT can evaluate scientific claims and highlight unresolved research questions which can in turn act as an impetus for ongoing research [48].

Importantly, systematic reviews of generative AI tools in evidence synthesis show that major tasks such as literature searching and bias assessment still require human oversight, as models continue to produce false inclusions, omissions, and inconsistent interpretations without supervision.

### AI Assistance in Academic Writing

LLMs influence how scientific writing itself is produced and evaluated. A cross-sectional study published in *JAMA Network Open* compared medical research abstracts written by surgical trainees, senior surgeons, and ChatGPT. ChatGPT was given previously written, unassociated abstracts by the senior author, three papers on the surgical topic and the current study data after processed by a statistician. Blinded, very experienced, surgical reviewers were unable to reliably distinguish AI-generated abstracts from those authored by humans, and ChatGPT outputs scored comparably to resident and senior surgeon abstracts, demonstrating that LLMs can produce high-quality academic text when provided structured prompts and appropriate oversight [49].

In addition, recent reviews report researchers utilizing AI to streamline manuscript writing tasks. An analysis of ChatGPT in medical research describes its use for drafting and editing assistance, generating structured outlines, citation and reference support, and table or figure creation [50]. While these tools may streamline writing and editing tasks, they do not replace the need for domain expertise, accurate citation practices, and rigorous verification of all claims.

### LLM for Data Extraction and Audit

Extracting structured data from clinical notes remains a time consuming yet essential component of clinical research. LLMs have demonstrated the ability to convert unstructured EHR text into structured datasets for audit and research purposes, achieving reported accuracy rates of 90%–95% across a range of clinical tasks [51]. In Urology, ChatGPT extracted key variables from operative [52] and pathology [53] reports with high accuracy and generalizability. The ability of LLMs to rapidly process large volumes of text makes them particularly well suited to large-scale chart abstraction and have been used successfully to extract information from extensive medical records pertaining to breast cancer [54]. Beyond data extraction, generative AI tools such as Google Bard have been leveraged to develop algorithms that analyze EHR-derived Excel datasets, significantly improving the efficiency of surgical quality assurance audits by reducing reliance on manual data review [55].

### Transparency and Publication Guidelines

The academic community is actively developing ethical frameworks and reporting standards for use of LLMs in academic work. Existing guidance consistently emphasizes three core principles: 1) human authors must take responsibility and vet all AI outputs, 2) meaningful human intellectual contribution must be present, and 3) transparent acknowledgement of AI use should be included in publication submissions to support reproducibility, credibility, and research integrity [56]. Major medical publishers, including SAGE, Nature Portfolio, and Elsevier, generally permit limited use of LLMs for basic language editing tasks such as grammar, spelling, and punctuation, and such use typically does not require formal declaration. In contrast, these publishers prohibit the use of generative AI for peer review and editorial decision-making and caution against uploading full manuscripts or confidential review materials into external AI tools due to concerns regarding confidentiality, intellectual property, and data security.

Detection of LLM-generated text remains imperfect, and proposed methods include statistical approaches and experimental detection techniques; however, these strategies have limitations and are not uniformly adopted [57].

### Guidelines for AI Use in Academic Research

As AI becomes more ubiquitous in academic research, authors must retain responsibility for ensuring that the tone, reasoning, and intellectual framing reflect their own understanding of the subject matter. LLMs can enhance efficiency and support idea development, but their use must not compromise academic authenticity or accuracy.

Mijatović and colleagues have proposed practical guidance for the responsible use of AI in scientific writing, emphasizing the need to verify references and factual claims against primary sources and to critically edit LLM-generated text to ensure alignment with the author’s intent. These guidelines highlight that the research foundation and analysis should be the authors, with the LLM used as a support tool, not substitution for critical thinking. Individual institutional guidelines must be followed and it is recommended to discuss AI use with the collaborative research team and mentors to ensure safe use [58].

### Future Applications

The near-term research applications of LLMs will likely center on reducing friction in study execution rather than replacing analytic judgment. These uses may include automated eligibility screening from structured EHR data, semi-automated chart abstraction with human verification, and generation of standardized case report forms and data dictionaries to improve multi-site alignment. LLMs may also assist with literature surveillance and adverse-event narrative classification, but these workflows require prospective validation and clear audit trails to ensure reproducibility.

## Discussion

As AI use becomes widespread in medicine, surgeons need to weigh potential technological, workflow benefit against the inherent risks of probabilistic language systems. This technology still requires close human oversight. It is still imperative to fact check LLM output and patient privacy must be protected. The limitations discussed highlight the key technical, ethical, and jurisdictional considerations fundamental to safe and effective LLM use in surgical practice.

### Prompting (Prompt Engineering and Context Management)

The effectiveness of LLMs is highly dependent on the quality of the prompts provided to the generative AI. To provide an output, the LLM tokenizes individual prompt words or parts of words, which are then assigned a unique numerical value. These numerical tokens are fed into the model, and the quality of the output relies on the complexity of the artificial neural network. The program constructs its response based solely on pattern recognition and does not understand the meaning of the words [59]. Well-constructed prompts help align the AI system to generate clinically relevant and accurate outputs. Clear, specific, and context-appropriate prompts are essential. The following principles may help surgeons optimize their use of LLMs [60].

1. Define the objective. Clearly stating the intended goal of the LLM. Specify the clinical task, the intended audience, and the desired output format.
2. Provide explicit and specific instructions, including role assignment to align the LLM with appropriate clinical expertise (e.g., “assume the role of an experienced surgical attending”).
3. Define contextually relevant parameters applicable to the clinical scenario.
4. Employ iterative refinement, using a structured feedback loop guided by clinical judgment.
5. Align outputs with evidence-based practice. Instruct the LLM to review literature such as reference guidelines or key studies. AI-generated outputs require independent verification to ensure accuracy and avoid hallucinated content [61].

#### Management of Protected Health Information

A principal advantage of this technology is the ability to recall prior use interactions, enhancing the accuracy and relevance of subsequent outputs [62]. This function enables surgeons to refine and optimize their prompts, facilitating the generation of more precise, clinically relevant responses. This beneficial capability requires caution in the clinical setting, particularly with respect to Protected Health Information (PHI). PHI entered into non-approved systems may be retained or reproduced in subsequent outputs depending on platform behavior and settings [63]. Surgeons should restrict generative AI use to scenarios that do not require entry of PHI, and should use only institutionally approved, HIPAA-aligned tools (e.g., EHR-integrated solutions) when patient data are involved.

#### Hallucinations

The accuracy of information is very important in clinical applications. LLM can produce inaccurate or fabricated information, known as ‘hallucinations”, resulting from an output being incoherent or misrepresentative of the source and are generally due to errors in coding and decoding. These discrepancies can arise in AI-generated responses as users lack visibility into the underlying source material from which specific outputs are derived [64]. Although the time-saving potential of LLMs is appealing in a time-constrained profession, particularly for automating repetitive tasks like discharge summaries, these applications still require close clinician oversight to ensure accuracy and mitigate harm. A study published in *JAMA* Internal Medicine reported that errors generated during LLM-assisted discharge summaries generally carried low potential for patient harm, but physician review remain necessary for safety and compliance [34].

Within surgical research, hallucinated citations are a well described failure mode. LLMs may fabricate references and inappropriately misattribute claims to nonexistent or unrelated publications [59, 65]. This risk is especially consequential in academic work and requires careful verification of all references and claims.

While LLMs are trained on large text corpora, the lack of transparency surrounding their training data necessitates caution. Clinicians should consider patient demographics carefully, as generalized outputs may not reflect the needs or characteristics of individual or underrepresented populations [66]. Implicit bias encompasses unconscious negative or positive stereotypes that unintentionally influence judgments, decisions, and actions; and can be present within the LLMs response as these are trained on datasets arising from internet and can reflect the common biases in culture [59].

#### Liability

The allocation of responsibility for errors and their potential impact on patient care needs to be considered closely. Due to their complexity and limited foreseeability, AI systems are not easily accommodated within traditional liability frameworks [67]. The delineation of responsibility among stakeholders in medical liability cases is complicated when AI systems are incorporated into clinical decision-making. As this technology increasingly mediates the relationship between clinician actions and patient outcomes, establishing causation, negligence, or harm becomes more challenging for patients [68]. Notably, public perceptions of liability differ from those of clinicians. Members of the public are significantly more likely than physicians to believe that physicians should be held responsible for errors occurring during AI-assisted care [69]. Without a clear framework for liability associated with AI in healthcare, surgeon hesitation is reasonable.

## Conclusion

As AI adoption accelerates, prioritizing accuracy, privacy, and patient safety remains essential. Although LLMs can assist with multiple clinical and academic tasks, safe use currently requires consistent human oversight and verification. Surgeons should view LLMs as assistive tools rather than autonomous clinical agents, applying structured prompting, rigorous fact-checking, and privacy-conscious workflows while maintaining human judgment and accountability.
